# Optimizing Sow and Litter Performance via a Comprehensive Service-to-Weaning Feeding Regimen

**DOI:** 10.3390/ani15192821

**Published:** 2025-09-27

**Authors:** Julia Cantin, Carlos Cantin, Olga Mitjana, Maria Teresa Tejedor, Carlos Gil-Rubio, Ana Maria Garrido, Maria Victoria Falceto

**Affiliations:** 1 Department of Animal Pathology, Faculty of Veterinary Sciences, University of Zaragoza, 50013 Zaragoza, Spain; jcantin@unizar.es (J.C.); amgarrido@unizar.es (A.M.G.); vfalceto@unizar.es (M.V.F.); 2 Independent Researcher, 50009 Zaragoza, Spain; carlos@agnporcino.com; 3 Agroalimentary Institute of Aragon-IA2, University of Zaragoza–Centro Investigación Tecnología Agroalimentaria de Aragón (CITA), 50013 Zaragoza, Spain; 4 Department of Anatomy, Embryology and Animal Genetics, Faculty of Veterinary Sciences, University of Zaragoza, 50013 Zaragoza, Spain; ttejedor@unizar.es; 5 Centro de Investigación Biomédica en Red Enfermedades Cardiovaculares (CIBER CV), Faculty of Veterinary Sciences, University of Zaragoza, 50013 Zaragoza, Spain; 6 Veterinary Technical Service Nutega, CCPA Group, 28823 Coslada, Spain; carlosgil.vet@gmail.com

**Keywords:** hyperprolific sow, beta-hydroxybutyrate, diets, productivity

## Abstract

Hyperprolific genetic lines achieve large litters but are at increased risk of metabolic stress, impaired sow condition, and reduced piglet viability. This study aimed to evaluate whether a phase-specific feeding program from service to weaning improves sow metabolic status and body condition, and enhances offspring performance, compared with a standard program. Sixty gilts and 268 multiparous sows were randomly allocated to a control group (standard commercial diets; C) or a treatment group (phase-specific diet; T) covering early and late gestation, peripartum, and lactation. Compared to a commercial feeding strategy, four custom-made, phase-specific diets were fed as follows: for early gestation (greater amount of essential aa and fat content), late gestation (higher amount of CP and essential aa), peripartum (improved nutrient profile at lower fat and fiber content), and lactation (higher essential aa and calorie content). On gestation day 113, T gilts had greater backfat thickness (BFT) and lower blood β-hydroxybutyrate (BHBA) than C. T multiparous sows showed greater BFT and longissimus muscle depth (LMD) and lower BHBA on day 113, fewer stillborn piglets, lower incidences of neonatal diarrhea, and fewer negative lactation curves and postpartum hypophagia. On day 28 of lactation, T litters had heavier piglets, and LMD remained higher in T sows. The phase-specific feeding program improved the metabolic state, body condition, and reproductive outcomes in hyperprolific sows and enhanced offspring growth through weaning.

## Simple Summary

Hyperprolific sows can face metabolic challenges during pregnancy and lactation that affect their condition and the survival of their piglets. This study evaluated whether a feeding program adapted to each phase—from service to weaning—could improve health and performance. A total of 60 gilts and 268 multiparous sows were assigned to either a standard diet throughout the cycle or a tailored diet that changed across early and late gestation, the peripartum period, and lactation. Sows on the tailored program showed a better body condition and produced piglets that were healthier and heavier at birth and at weaning. They also had fewer stillborn piglets, fewer feeding problems after farrowing, and fewer cases of newborn diarrhea. Blood tests near the end of pregnancy indicated a better energy balance in the tailored-diet group. Overall, adapting the diet to each phase of the reproductive cycle supported sow health and improved the growth and well-being of their offspring.

## 1. Introduction

Productivity in swine farms largely depends on the reproductive outcomes of sows. Over the last three decades, genetic advancements have significantly increased sow prolificacy, with new lines capable of producing between 18 and 20 piglets per litter [1,2]. However, high prolificacy presents physiological challenges during gestation, farrowing, and lactation, especially as sow feed intake capacity has not increased proportionally [3]. This imbalance can lead to nutritional deficiencies affecting fetal development and causing metabolic issues in sows, resulting in low-birth-weight piglets, stillbirths, and increased neonatal mortality [4,5]. The increase in the number of piglets born is associated with a decrease in individual birth weight and greater within-litter variability in weight [5]. This variability impacts piglet survival, particularly if piglets cannot secure adequate energy from colostrum, a critical energy source on their first days of life [6]. In addition, while the sow’s milk production increases with litter size, the amount of milk available per piglet decreases [7]. Low-birth-weight piglets have less capacity to consume and digest colostrum, raising their risk of neonatal mortality [8].

To maximize reproductive efficiency and optimize productive outcomes in sows, it is essential to identify critical periods in the reproductive cycle and adjust dietary strategies accordingly. These key phases include from service to 50 days of gestation, from 51 to 110 days of gestation, from 111 days through the peripartum period, and from peripartum through lactation. During early gestation, specific nutrients play crucial roles in embryonic development, maternal recovery, and embryonic establishment. As is known, increasing dietary fat content enhances net energy intake, facilitating the restoration of body condition post-lactation [9]. Furthermore, previous studies have shown that arginine supplementation improves embryonic implantation and placental development, ultimately increasing embryo survival and the number of live-born piglets [10,11]. It is also known that methionine supports essential cellular functions, including oxidative stress management and vitamin B metabolism, which are critical during early gestation [12].

Nutritional interventions during mid-gestation focus on optimizing fetal growth and maternal health. Increasing dietary amino acid intake improves uterine muscle tone, reducing the incidence of dystocia and stillbirths [13,14]. Higher fiber levels toward the latter stages of gestation contribute to lower neonatal mortality, enhanced intestinal integrity, and reduced digestive disorders in piglets [15]. The peripartum phase requires targeted nutritional adjustments to support parturition and colostrum production. As fetal growth and colostrum synthesis demand increased energy, deficits in energy and lysine intake are associated with higher stillbirth rates and decreased milk quality during lactation [16]. Calcium intake is essential for fetal skeletal development and the prevention of hypocalcemia in sows, which can contribute to farrowing complications and increased neonatal mortality [17]. Additionally, sufficient NDF (neutral detergent fiber) levels in the peripartum diet help maintain intestinal transit and stabilize blood glucose levels, facilitating labor and mitigating dystocia risks [16].

During lactation, sows experience a substantial rise in nutritional requirements to sustain milk production and maintain body condition. A sufficient intake of energy and protein is necessary to meet the increased metabolic demands of lactation and maternal recovery from gestation [13,18]. Lysine is crucial for mammary gland metabolism and high-quality milk production, while calcium and phosphorus are required to restore maternal bone mineral reserves and support lactation performance [19]. A strategic approach to sow nutrition, addressing specific needs across the reproductive cycle, is essential for optimizing reproductive performance, enhancing piglet survival, and improving overall herd productivity.

One of the primary metabolic challenges in hyperprolific sows is ketosis, a condition that arises when a glucose or amino acid deficit triggers the body to oxidize fatty acids for energy, producing ketone bodies such as β-hydroxybutyrate (BHBA). Ketosis can cause appetite loss and poor body condition [20,21], and the process occurring amid ketosis may lead to excessive lipolysis and triglyceride accumulation in the liver, known as fatty liver, which deteriorates the sow condition and reproductive capacity in the next cycle [22]. To prevent these issues, additional energy sources and enhanced amino acid profiles in the diet are recommended [23,24]. Supplementing arginine, methionine, and lysine at each stage of gestation and lactation improves fetal development, piglet birth weights, and milk quality [25]. Additionally, fiber in the diet helps stabilize sow metabolism, supports intestinal transit, and reduces the risk of dystocia and stillbirths [15].

This study aims to analyze changes in the nutritional parameters of hyperprolific sows in order to approximate the levels of essential nutrients at each stage of gestation and lactation. The research focuses on the effects of these nutrients on sow body condition, metabolic state, and the number and weight of live-born and weaned piglets. It has been proposed that providing additional energy and amino acid supplementation during the peripartum phase may reduce stillbirths in multiparous sows, since this period is characterized by markedly increased nutritional demands [26,27].

## 2. Materials and Methods

All protocols used in this study were approved by the Animal Ethics Committee of the University of Zaragoza (reference number: PI37/23). The procedure was carried out on a commercial farm in north-eastern Spain. Expert veterinarians supervised the care and handling of the animals and ensured their welfare throughout the experiment.

### 2.1. Experimental Design, Housing, and Management

A total of 328 females (268 multiparous sows and 60 gilts, Landrace × Large White) from the hyperprolific DanBred genetic line were randomly and equally assigned to control (C) and modified diet (T) groups. A randomized block design was used; treatments were applied at the individual sow level within rooms separated by group; and the sow was defined as the experimental unit. No pen/season/potential confounding effects were accounted for in the experimental design.

Table 1 shows the distribution of these multiparous sows in terms of cycles and groups. The sows in cycles 6, 7, and 8 were grouped as ≥6 cycles for the statistical analyses.

**Figure 1:**
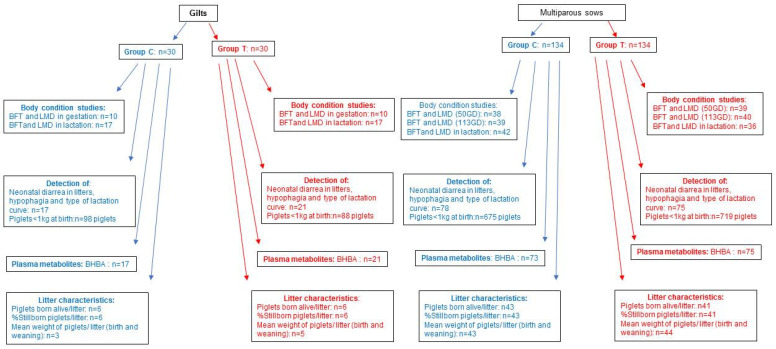
Flow diagram presenting the animals recruited and finally analyzed. BFT: Backfat thickness; LMD: loin muscle depth; BHBA: ketone bodies in blood.

The inclusion criteria were healthy, pregnant females (ultrasound diagnosis at 23 days) with a body condition of 3 (3/5), assessed using a visual score [3]. Throughout the study, some health problems arose that prevented data collection from all the individuals initially recruited; data from affected animals were discarded so as not to bias the study results. Figure 1 shows a flow diagram presenting the animals recruited and finally analyzed.

During gestation, the sows and gilts were housed separately in pens (3 m^2^/animal with a partially slatted floor), during the period of gestation. The temperature and humidity were maintained within a controlled range, with a target of 16–22 °C and 60–70%, respectively. Feeding was carried out by means of a semi-box system with dispensers, and each pen was equipped with 5 cup-type drinkers.

From day 110 of gestation to day 28 of lactation, the sows were housed individually in farrowing crates (1.80 × 2.65 m) within a room maintained at a temperature of 18–22 °C and a humidity level below 65%. Each sow was provided with a dedicated water trough (3 L/min flow rate) and received 5 L of water per feeding. The farrowing pens included a separate piglet rearing area with heating and an infrared lamp to maintain a temperature of ~30 °C. The litter size was equalized to 13–14 piglets per litter within 24 h post-farrowing in both groups. Cross-fostering was performed to homogenize litter size (13–14 piglets per sow) within the first 24 h postpartum, following a standardized protocol. It was applied equally to both groups under the same conditions. Afterwards, during lactation, no further piglet movements were allowed.

### 2.2. Dietary Treatments, Feeding, and Feeding System

Two feeding diets were designed and randomly assigned to the control group (C) and the treatment group (T) in this experiment (see Table 2).

#### 2.2.1. Diet of Control Group

The gestational feed was provided to the sows from weaning to day 110 of gestation (gestation standard), after which a single lactational feed was administered from day 111 of gestation to day 28 post-partum (lactation standard). The two feeds were standard commercial diets, formulated in accordance with the recommendations set forth by FEDNA (Fundación Española para el Desarrollo de la Nutrición Animal) for production sows [27].

#### 2.2.2. Modified Diet Group

Sows in the treatment group (T) were managed with a phase-adapted feeding strategy, in which diet composition and daily allowance were adjusted to each stage of the reproductive cycle. This strategy consisted of four phase-specific feeds, detailed in Table 2.

Gestation 1 (service to day 50): the feed was formulated to enhance the digestible arginine/lysine ratio (+53%), fat (+25%), and digestible methionine (+0.085%).

Gestation 2 (days 51–110): the feed was enriched with crude protein (+0.86%) and digestible lysine (+0.092%).

Peripartum (day 111 of gestation to day 3 postpartum): the feed included increased crude protein (+4.71%), lysine (+0.362%), and calcium (+0.1%), while reducing fat (–0.854%) and crude fiber (–2.492%). It also contained a glycogenic precursor and at least 6% beet pulp to improve energy supply and intestinal function.

Lactation (day 4 postpartum to weaning): the feed was formulated with enhanced amino acid profiles, increased lysine (+0.068%), and higher metabolizable energy (+26.242 kcal/kg), supplemented with 5% full-fat soy to improve digestibility and palatability. Both groups received a lactation feed with an electrolyte balance of approximately 200 (meq/kg).

### 2.3. Feeding: Feed Curve and Number of Daily Intake

During gestation, group C was managed with a U-shaped feeding curve, while group T followed a flat feeding curve. In practical terms, the flat curve (group T) meant that gilts received a constant allowance of 2.5 kg/day from service until farrowing, regardless of the stage of gestation.

In contrast, the U-shaped curve (group C) involved adjusting the daily ration according to the stage of pregnancy: higher immediately after service, restricted during mid-gestation, and increased again in late gestation. For example, multiparous sows in group C consumed 3 kg/day from service to day 40, 2.2 kg/day from day 40 to 90, 3 kg/day from day 90 to 110, and 3 kg/day (in two meals) from day 110 until farrowing.

**Figure 2:**
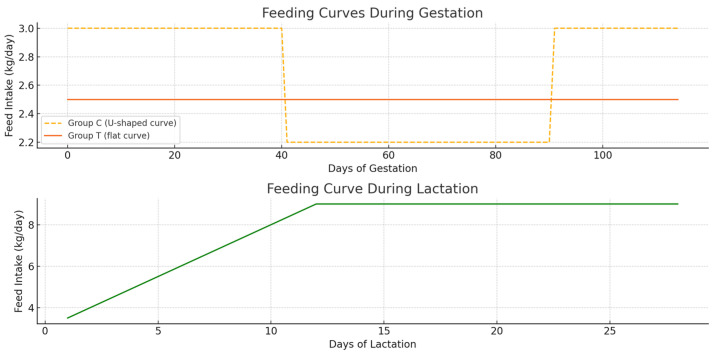
Feeding curves.

During the lactation and weaning period, all sows showed a similar feeding curve, starting with 3.5 kg/day on day 1 and increasing by 0.5 kg/day until reaching 9 kg/day on day 12. This level was maintained until day 28 of lactation. The sows received two meals per day until day 7, after which they received three meals per day. During the weaning–farrowing interval, the multiparous sows in both groups consumed 4 kg/day in two feedings (see Figure 2).

### 2.4. Body Condition, Hypophagia, and Lactation Curve

Body condition was assessed by measuring backfat thickness (BFT) and loin muscle depth (LMD) at the P2 reference point. The P2 point was defined as 6.5 cm lateral to the dorsal midline at the level of the last rib, using a wireless ultrasound scanner (Backfat & Loin Depth Scanner SF-1, SonicVet, Beijing, China) with a 5 MHz linear digital probe connected to an iPad via Wi-Fi. These measurements were carried out on all animals in both groups on days 50 and 113 of gestation, as well as on day 28 of lactation.

Clinical hypophagia (HP) was visually observed during the first lactation week. Sows were considered positive for HP if they failed to consume their ration for two consecutive feedings; they were considered negative if they consumed their ration.

The lactation curve was also examined visually throughout lactation. Lactation failure was recorded for sows that could not complete lactation and had to be removed from the farrowing pen due to insufficient milk production and an inability to adequately feed their litter. A negative lactation curve was defined as a litter weight significantly below the average with weight heterogeneity greater than 50%; conversely, a positive lactation curve indicated normal or above average litter performance.

### 2.5. Sow Plasma Metabolites

To determine the presence of ketone bodies in blood, β-hydroxybutyrate (BHBA, mmol/L) was measured using the Freestyle Precision β-Ketone system with B-ketone test strips (Abbott Laboratories, Chicago, IL, USA). Blood samples were collected from the coccygeal vein at 113 days of gestation, between 08:00 and 10:00 h, prior to the morning feeding, in order to avoid postprandial variation. Samples were allowed to clot at room temperature and centrifuged at 1500× *g* for 15 min to obtain serum. Each sample was analyzed in triplicate, and the mean of the three measurements was used for statistical evaluation. For classification purposes, sows with a BHBA concentration equal to 0 mmol/L were considered negative, whereas values > 0 mmol/L were considered positive [22].

### 2.6. Litter and Piglet Production Parameters

Farrowing was strictly monitored from start to finish in order to record the total number of piglets born (TB), including those born alive (BA) and stillborn (SB). The SB were recorded by direct observation during farrowing, classified as piglets born dead with fetal membranes, friable tissues, sometimes already showing decomposition, and hooves covered with a yellowish slipper-like skin. Low-birth-weight piglets were identified through the individual weighing of live piglets before colostrum intake, with <1 kg defined as the threshold.

At the time of birth, piglets from 6 gilts and 43 multiparous litters in group C, and from 6 gilts and 44 multiparous litters in group T were weighed individually prior to the intake of colostrum. Piglets weighing less than 1 kg were identified as having intrauterine growth retardation (IUGR) [28,29]. At 28 days, piglets from 3 gilts and 43 multiparous litters in group C, and from 5 gilts and 44 multiparous litters in group T were weighed again. The litters weighed at weaning corresponded to those weighed at birth within each group.

Litters with diarrhea during the first week of lactation were visually counted, being considered positive with clinical symptoms from the first piglet with diarrhea [30] and negative, when the litter did not present diarrhea.

### 2.7. Statistical Methods

A statistical analysis was performed using SPSS v 26 software (IBM, Chicago, IL, USA). Qualitative variants are shown as percentages. The normality of quantitative variables was tested using the Shapiro–Wilk test and graphical methods (Q-Q plot). Variables with an approximately normal distribution are summarized using mean and standard deviation. For BHBA, a strongly discontinuous variable not normally distributed, median and interquartile range (IQR) are used; minimum and maximum values are also shown.

The percentages were compared between groups using Pearson’s chi-squared (with continuity correction for 2 × 2 tables) or alternatively using Fisher’s exact test. For normal quantitative variables, ANOVA was applied. In the gilts, the models included only group (C or T, fixed effect) for unique measurements (one way ANOVA) or group and time (birth and weaning) as fixed effects and interaction for repeated measurements (two-way mixed ANOVA). In the multiparous gilts, group, cycle (fixed effects in a two-way ANOVA), and interactions were considered for unique measurements, while group, cycle, time (fixed effects), and all possible interactions were included for repeated measurements (three-way mixed ANOVA). No covariates were available for the gilts or multiparous gilts. For BHBA, the Mann–Whitney U test was applied for comparisons between treatments. The Spearman correlation (r_s_) was used to assess potential relationships between BHBA and LMD, BHBA and BFT, and BHBA and litter size. The relationships between BFT and LMD and between litter size and these variables were evaluated using the Pearson correlation (r).

In every case, *p* values < 0.05 were considered statistically significant. When significant differences were found for more than two comparison terms, multiple comparisons with Bonferroni correction were applied.

## 3. Results

Unfortunately, there were technical and farm management reasons for not being able to take data from all sows used in the study. Some data were missing due to a health issue on the farm; affected sows had to be removed from the study population to avoid incongruent results.

### 3.1. Body Condition

**Figure 3:**
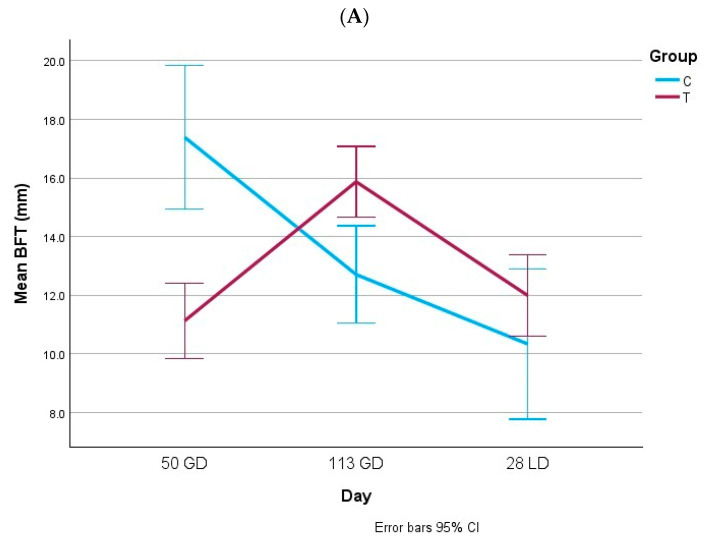

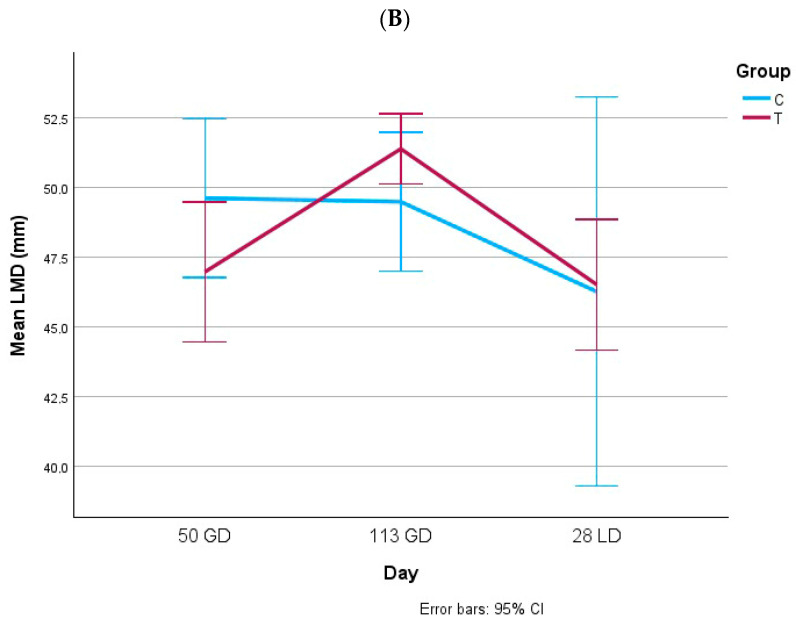
Backfat thickness (mm, (**A**)) and loin muscle depth (mm, (**B**)) in gilts. BFT: backfat thickness; LMD: loin muscle depth; GD: gestation day; LD: lactation day. (**A**) 50 GD: *p*_group_ < 0.001; 113 GD: *p*_group_ = 0.003; 28 LD: *p*_group_ = 0.169. (**B**) 50 GD: *p*_group_ = 0.131; 113 GD: *p*_group_ = 0.143; 28 LD: *p*_group_ = 0.922.

**Figure 4:**
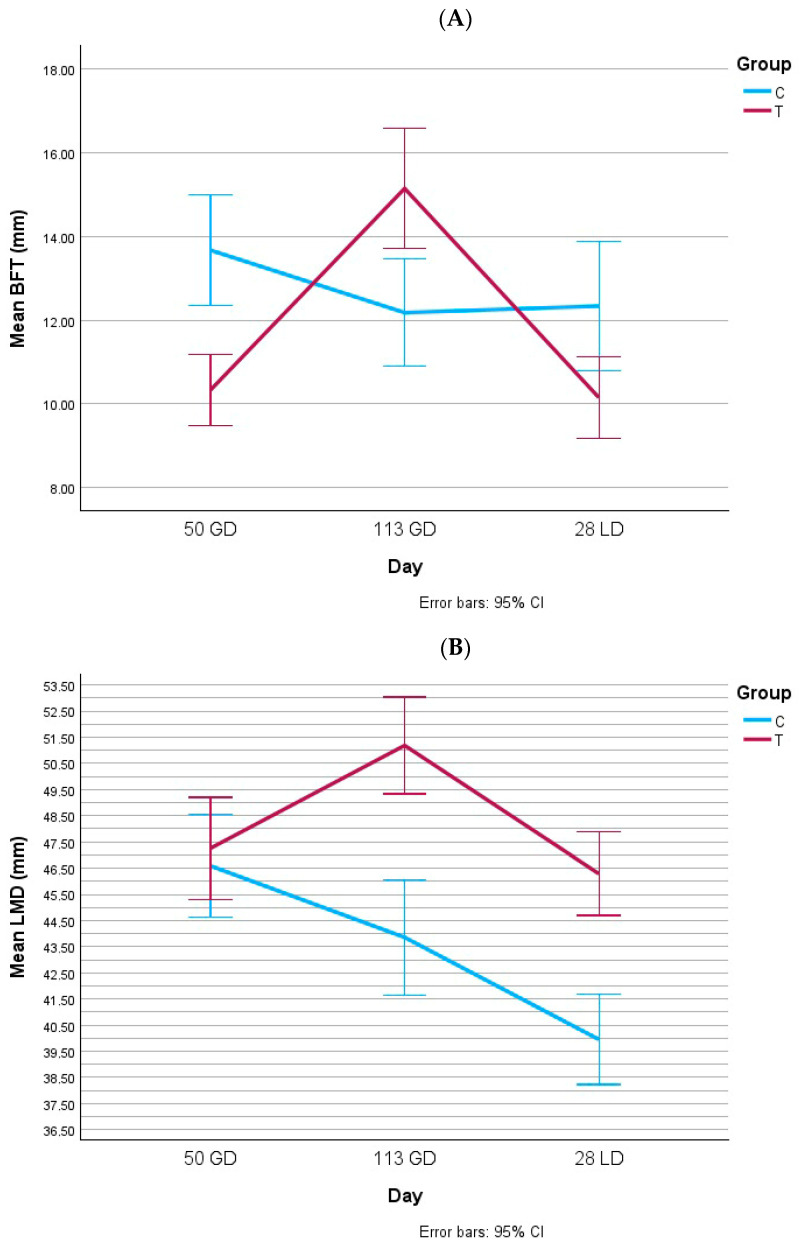
Backfat thickness (mm, (**A**)) and loin muscle depth (mm, (**B**)) in multiparous sows. BFT: backfat thickness; LMD: loin muscle depth; GD: gestation day; LD: lactation day. (**A**) 50 GD: *p*_group_ < 0.001; 113 GD: *p*_group_ = 0.014; 28 LD: *p*_group_ = 0.127. (**B**) 50 GD: *p*_group_ = 0.976; 113 GD: *p*_group_ < 0.001; 28 LD: *p*_group_ < 0.001.

Figure 3 and Figure 4 summarize the body condition measurements. On gestation day 50, BFT was higher in both gilts and multiparous sows from group C, while no group differences were found for LMD. By gestation day 113, BFT was higher in both gilts and multiparous sows from group T. In multiparous sows, LMD was also significantly higher in group T at this stage. At lactation day 28, no differences in BFT or LMD were found in gilts, whereas multiparous sows in group T maintained significantly higher LMD values. Overall, the adjusted feeding strategy improved the body condition in late gestation and preserved muscle depth during lactation in multiparous sows.

### 3.2. Occurrence of Diarrhea, Hypophagia, Positive Lactation Curve, and Low Weight Piglets

**Figure 5:**
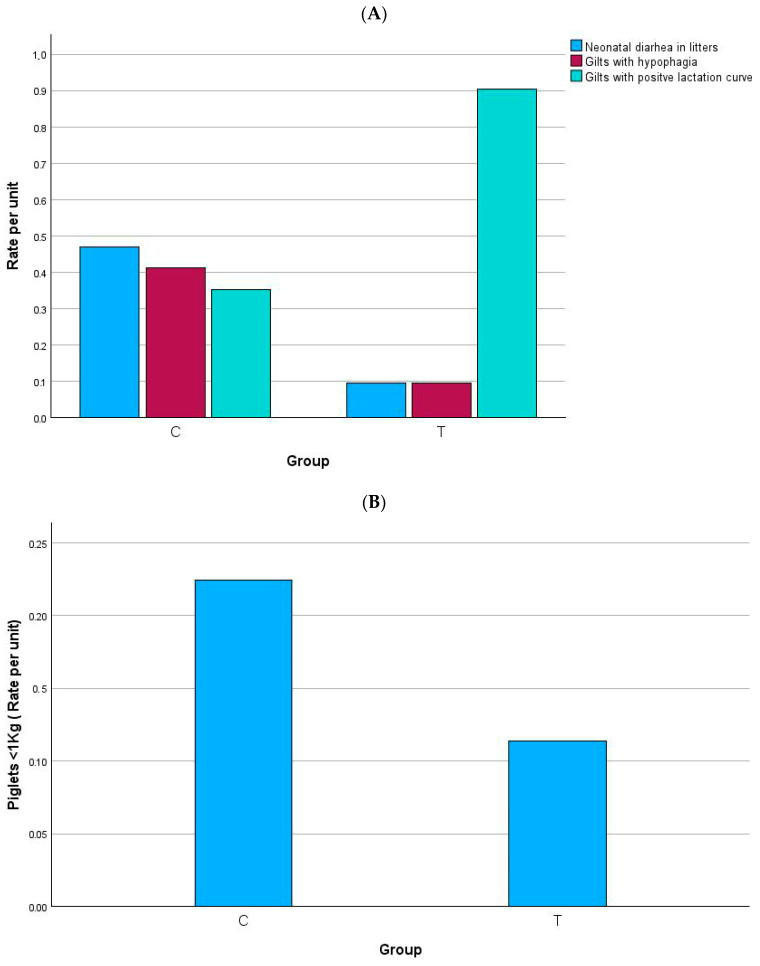
Occurrence (rate per unit) of neonatal diarrhea, hypophagia and positive lactation curve (**A**), and low-birth-weight piglets (**B**) in gilts. Comparisons between groups were carried out by using Pearson’s chi-squared or Fisher’s exact test. (**A**) Neonatal diarrhea in litters: *p* = 0.023; gilts with hypophagia: *p* = 0.028; gilts with positive lactation curve: *p* = 0.001. (**B**) *p* = 0.035.

**Figure 6:**
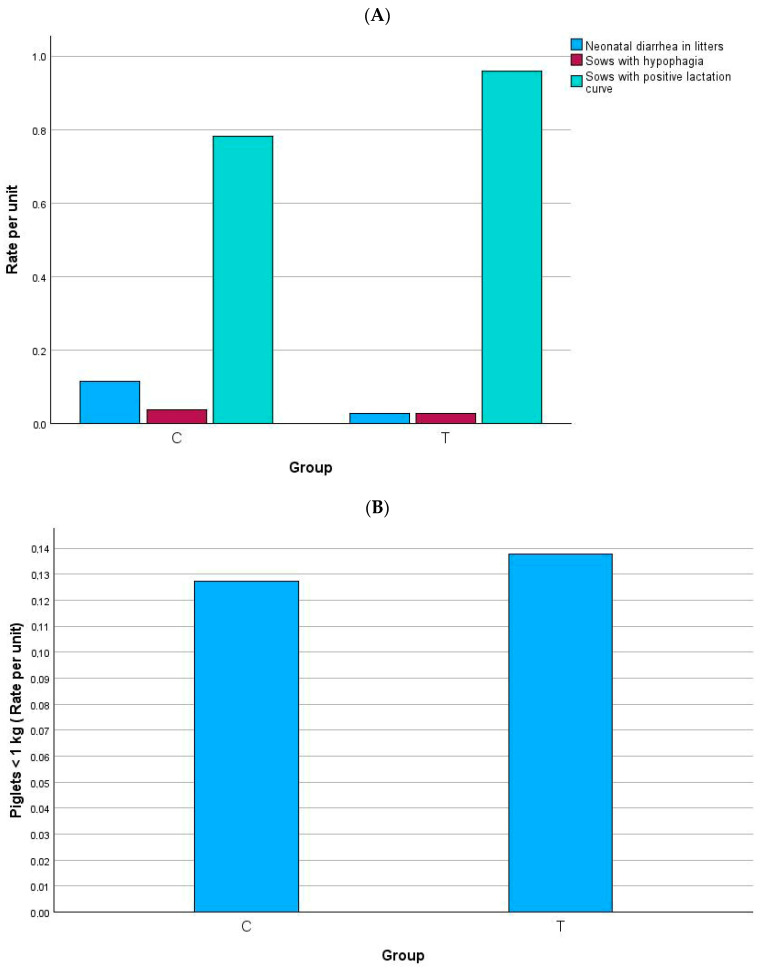
Occurrence (rate per unit) of neonatal diarrhea, hypophagia and positive lactation curve (**A**), and low-birth-weight piglets (**B**) in multiparous sows. Comparisons between groups were carried out by using Pearson’s chi-squared or Fisher’s exact test. (**A**) Neonatal diarrhea in litters: *p* = 0.033; gilts with hypophagia: *p* = 1.000; gilts with positive lactation curve: *p* = 0.002. (**B**) *p* = 0.627.

Figure 5 and Figure 6 show the main outcomes. Diarrhea in the first week was significantly more frequent in group C in both gilts and multiparous sows. Postpartum hypophagia was significantly lower in group T gilts but did not differ between groups in multiparous sows. Regarding the lactation curve, both gilts and multiparous showed a significantly higher proportion of positive curve in group T. In gilts, the percentage of low-birth-weight piglets (<1 kg) was nearly halved in group T compared with group C. No overall differences were observed in multiparous sows. These results indicate that the experimental diet improved piglet viability and sow lactation performance, particularly in gilts.

### 3.3. Sow Plasma Metabolites

As shown in Table 3, BHBA concentrations on gestation day 113 were significantly higher in group C in both gilts and multiparous sows. Differences were particularly evident in cycles 3 and ≥6 of multiparous sows. In both gilts and multiparous sows, BHBA was negatively correlated with both LMD and BFT. In multiparous sows, LMD and BFT showed a positive correlation.

No significant correlation for litter size was detected in any case (Table 4). This confirms a closer association between elevated BHBA levels and body reserve mobilization in late gestation.

### 3.4. Litter Characteristics

Table 5 presents litter outcomes. In gilts, no group differences were observed for piglets born alive/litter or stillbirth percentage/litter. After correcting for cycle, in multiparous sows, the group effect was not significant for piglets born alive/litter, but the percentage of stillbirths/litter was lower in group T. Thus, the experimental feeding program contributed to reducing stillbirths in multiparous sows.

### 3.5. Weight of Piglets at Birth and at Weaning

**Figure 7:**
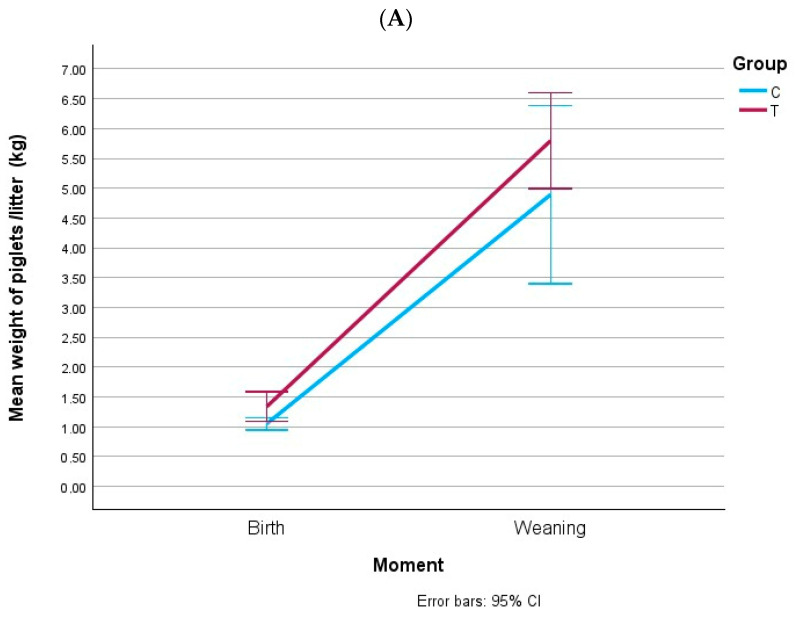

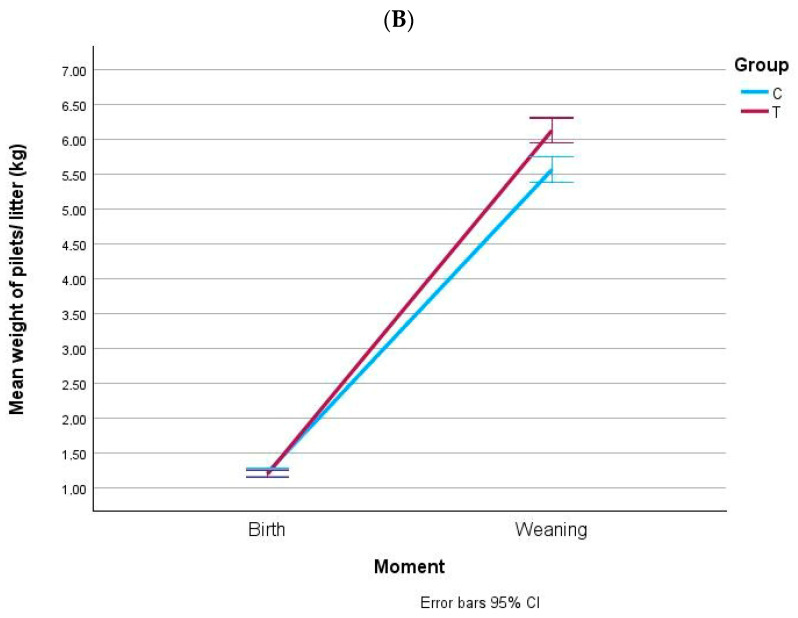
Mean weight of piglets/litter at birth and weaning from gilts (**A**) and multiparous sows (**B**). BFT: backfat thickness; LMD: loin muscle depth; GD: gestation day; LD: lactation day. (**A**) *p*_group_ = 0.068. (**B**) Birth: *p*_group_ = 0.613; weaning: *p*_group_ < 0.001.

Figure 7 shows piglet weights’ evolution. In gilts, piglet weight increased from birth to weaning in both groups, with no effect of diet. In multiparous sows, piglet weight at birth did not differ between groups, but at weaning piglets from group T litters were significantly heavier (*p* < 0.001). Therefore, the adjusted diet improved piglet growth during lactation in multiparous sows.

## 4. Discussion

### 4.1. Maternal Condition

The adjusted feeding strategy in the experimental group, which included 14% more feed during mid-gestation and 20% less in late gestation, was associated with better preservation of body condition (BFT and LMD) in both primiparous and multiparous sows. This pattern can be explained by the higher availability of amino acids and energy during critical phases of fetal and maternal development, which reduced excessive mobilization of body reserves and the risk of hypophagia during early lactation. These findings are consistent with previous reports showing that additional protein and energy improve metabolic integrity and prevent catabolism [14,31,32,33,34,35,36]. However, the feeding scheme used in this trial provides a novel approach, as it demonstrates that a redistribution of nutrients across gestation—not only supplementation—can be effective in maintaining sow condition. Overall, the nutritional strategy helped maintain the maternal condition without compromising the metabolic transition into lactation, which is critical in hyperprolific genotypes [3,18,34].

### 4.2. Parturition Failures/Performance

Control sows exhibited higher BHBA levels, indicating a more pronounced catabolic state and increased risk of ketosis around farrowing. Supplementation with energy and amino acids in the experimental group seemed to mitigate this condition by improving NEFA metabolism and glucose homeostasis [20,21,23]. This agrees with studies highlighting that optimizing maternal energy balance in late gestation reduces metabolic stress at farrowing [16,34,37,38].

In addition, the reduction in stillbirths observed in multiparous sows could be linked to a better energy and mineral balance during the peripartum phase, as energy or calcium deficits have been associated with prolonged parturition and increased neonatal mortality [15,34,37,38,39]. Sows with an insufficient supply of energy at farrowing often experience weaker uterine contractions and longer farrowing duration, which increases the likelihood of fetal asphyxia and intrapartum death. Conversely, maintaining adequate nutrient availability supports more efficient uterine activity and reduces parturition length, leading to improved piglet vitality.

Other authors have also emphasized that nutritional imbalances during the transition period compromise calcium homeostasis and oxidative balance, exacerbating dystocia and postpartum complications [40,41,42]. The fact that multiparous sows in the experimental group benefited more clearly suggests that these animals, with higher milk yields and body condition variability, may be especially sensitive to adjustments in dietary supply.

From a practical perspective, preventing farrowing complications is not only crucial for piglet survival but also for sow welfare and reproductive longevity. Difficult farrowings often require human intervention, which increases labor costs, raises the risk of infection, and may negatively affect subsequent reproductive cycles. Therefore, nutritional strategies that reduce peripartum stress represent a valuable management tool, particularly in commercial farms where hyperprolific sows face greater metabolic demands and a higher baseline risk of stillbirths. Thus, the adjusted diet during late gestation and peripartum supported a more efficient farrowing process with a lower risk of complications. These results emphasize the importance of fine-tuning nutritional strategies not only for fetal growth, but also for ensuring smooth parturition dynamics in hyperprolific sows, where the combination of large litter sizes and prolonged farrowing poses a significant reproductive challenge [40,41,42].

### 4.3. Piglet Performance

In the experimental group, a lower proportion of low-birth-weight piglets and a reduced incidence of neonatal diarrhea were observed, suggesting a positive role of specific amino acids (arginine, methionine) and improved colostrum quality. These results agree with studies reporting that such nutrients enhance placental angiogenesis, passive immunity, and fetal muscle development [4,10,11,12,24,25,43,44,45,46]. Moreover, the better maternal body condition and optimized lactation curves translated into higher weaning weights, which is consistent with evidence that maternal nutritional status directly influences piglet viability and growth [5,6,28,29,30,44,46]. In summary, the nutritional strategy improved both neonatal survival and postnatal growth, reducing reproductive failures commonly associated with hyperprolific sows. This highlights the potential of targeted feeding interventions to mitigate the challenges of large litter sizes and to improve both short- and long-term outcomes in piglets [1,2,7,8,47,48].

### 4.4. Limitations

This study was conducted under controlled conditions with a limited number of animals, which restricts the direct extrapolation of the results to commercial farms with greater management variability. Under these conditions, the number of animals finally considered even decreased due to management and health problems on the farm. In addition, other biomarkers (e.g., hormonal or immunological parameters) were not evaluated, which could have provided further insight into the underlying mechanisms [19,22,49,50,51]. Future research should integrate these variables and validate the findings in diverse production systems [13,14,26,27,52]. Despite these limitations, the study provides solid evidence that adjusting feed quantity and composition is a practical tool to improve the health and performance of hyperprolific sows and their offspring. Importantly, the results underline the necessity of adapting feeding programs to modern sow genotypes, which present higher metabolic demands than traditional lines [9,17,33,35,53].

## 5. Conclusions

Adapting the diet to each phase of the breeding cycle improves the metabolic state and body condition of hyperprolific sows, especially multiparous sows, thereby benefiting the development of their offspring. Dietary modifications in group T significantly reduced the incidence of low birth weight in gilts and increased weaning weights in multiparous sows, and decreased diarrhea in litters and metabolic issues, particularly in gilts.

These findings highlight the importance of tailored nutrition to meet the specific needs at each stage of the reproductive cycle, and they suggest that further research on adapted diets is necessary to optimize outcomes.

## Figures and Tables

**Table 1:** Cycles and group distribution of multiparous sows.

Cycle	Group C	Group T	Total
1	6	3	9
2	32	16	48
3	24	16	40
4	20	26	46
5	19	36	55
6	12	27	39
7	20	10	30
8	1	-	1
Total	134	134	268

**Table 2:** Composition of the diet gestation standard and lactation standard of C group and composition of the diet gestation 1 feed, gestation 2 feed, peripartum feed, and lactation feed of T group.

Items (Units)	Gestation			Peripartum Feed (Group T)	Lactation	
	Gestation Standard (Group C)	Gestation 1 Feed (Group T)	Gestation 2 Feed (Group T)		Lactation Standard (Group C)	Lactation Feed (Group T)
**Ingredients:**						
Barley (%)	48.410	15.358	26.039	15.000	22.030	18.700
Maize (%)	0.000	24.976	25.000	24.971	19.440	30.076
Wheat (%)	0.000	10.800	0.000	19.700	0.000	11.200
Soybean meal (47% crude protein)	0.000	0.000	1.900	17.900	18.900	15.800
Animal blended fat (%)	0.000	2.450	0.000	0.000	0.000	0.000
Beet pulp (%)	0.000	10.000	5.000	6.000	0.000	5.100
Full fat soya (%)	0.000	0.000	0.000	0.000	0.000	5.000
Rice cylinder (%)	10.000	0.000	0.000	0.000	5.000	0.000
By-product biscuit (%)	0.000	0.000	0.000	0.000	7.000	0.000
Zootechnical meal (%)	7.430	0.000	0.000	0.000	10.000	0.000
Sodium bicarbonate (%)	0.100	0.000	0.000	0.000	0.310	0.000
Monocalcium phosphate (%)	0.090	0.000	0.000	0.000	0.580	0.000
Dicalcium phosphate (%)	0.000	0.610	0.890	1.060	0.000	1.035
Salt (%)	0.400	0.500	0.500	0.500	0.150	0.500
L-Lisine 50 (%)	0.330	0.130	0.426	0.351	0.270	0.337
L-Threonine (%)	0.040	0.000	0.070	0.025	0.050	0.050
L-Methionine (%)	0.000	0.042	0.065	0.000	0.000	0.000
Choline chloride (%)	0.050	0.000	0.000	0.000	0.040	0.000
Soybean oil (%)	0.500	0.000	0.700	1.127	1.480	2.352
Calcium carbonate (%)	1.620	0.625	1.110	0.000	1.720	1.350
Sunflower meal (36% crude protein)	10.000	9.210	10.000	5.066	2.700	0.000
Wheat bran (%)	18.980	25.000	25.000	5.000	10.000	8.300
Alfalfa (%)	0.480	0.000	0.000	0.000	0.000	0.000
Sepiolite (%)	1.320	0.000	0.000	0.000	0.000	0.000
Glucogenic precursor (%)	0.000	0.000	3.000	3.000	0.000	0.000
Others (%, additives) ^1^	0.240	0.300	0.300	0.300	0.300	0.300
**Nutritional analyses ^2^**						
Metabolizable energy (Kcal/kg)	2844.879	2935.790	2850.032	3049.971	3174.143	3200.385
Fat Matter (%)	4.106	5.138	3.440	3.252	5.008	5.502
Crude protein (%)	12.500	12.498	13.360	17.214	17.000	16.590
Crude fiber (%)	8.000	7.293	7.248	5.506	5.000	4.493
Neutral detergent fiber (%)	23.460	21.499	21.428	15.809	15.953	15.710
Arginine (%)	0.812	0.832	0.809	1.094	1.108	1.047
Digestible Arginine (%)	0.672	0.740	0.718	0.997	0.978	0.494
Lysine (%)	0.650	0.521	0.719	1.003	0.973	1.003
Digestible Lysine (%)	0.508	0.400	0.600	0.870	0.811	0.879
Methionine (%)	0.215	0.210	0.293	0.267	0.284	0.251
Digestive methionine (%)	0.176	0.179	0.261	0.235	0.243	0.221
Methionine + cystein (%)	0.479	0.472	0.579	0.589	0.587	0.569
Methionine + cysteine Digestive (%)	0.358	0.392	0.495	0.509	0.463	0.494
Calcium (%)	0.900	0.901	0.910	1.000	1.000	1.000
Phosphorus, Total (%)	0.616	0.567	0.638	0.584	0.613	0.575
Phosphorus, Digestive (%)	0.230	0.270	0.310	0.320	0.340	0.320
Electrolityc balance meq/Kg ^3^						200.000

^1^ Choline, phytases, antioxidants, mycotoxin sequestrant. ^2^ Calculated composition according to FEDNA [27]. ^3^ The dietary electrolyte balance was expressed as milliequivalents per kilogram (MEq/kg).

**Table 3:** Concentration of ketone bodies in blood (mmol/L) on gestation day 113 in both gilts and multiparous sows.

Age Group	Cycle	Group C				Group T				*p*-Value
		*n*	Min.	Max.	Median [IQR]	*n*	Min.	Max.	Median [IQR]	
Gilts										
	0	17	0.0	0.4	0.100 [0.1317]	21	0.0	0.1	0.00 [0.000]	0.016
Multiparous sows										
	1	5	0.0	0.3	0.100 [0.2]					
	2	15	0.0	0.2	0.000 [0.1]	9	0.0	0.0	0.000 [0.0]	0.108
	3	17	0.0	0.2	0.100 [0.1]	11	0.0	0.0	0.000 [0.0]	0.019
	4	12	0.0	0.1	0.050 [0.1]	14	0.0	0.1	0.000 [0.0]	0.131
	5	10	0.0	0.1	0.000 [0.1]	22	0.0	0.0	0.000 [0.0]	0.077
	≥6	19	0.0	0.3	0.000 [0.1]	19	0.0	0.0	0.000 [0.0]	0.025
	Global	73	0.0	0.3	0.000 [0.1]	75	0.0	0.1	0.000 [0.0]	<0.001

Min.: minimum observed value; Max.: maximum observed value; IQR: interquartile range.

**Table 4:** Correlation coefficients for concentration of ketone bodies in blood (mmol/L), loin muscle depth (mm), backfat thickness (mm), and litter size in both gilts and multiparous sows.

Age Group	Variable 1	Variable 2		
		LMD	BFT	Litter Size
		rs	r; rs	r; rs
		*p* Value	*p* Value	*p* Value
		*n*	*n*	*n*
Gilts	BHBA	−0.451	−0.529	−0.074
		0.046	0.016	0.820
		20	20	12
	LMD		0.116	0.257
			0.625	0.473
			20	10
	BFT			0.030
				0.935
				10
Multiparous sows				
	BHBA	−0.583	−0.345	0.004
		<0.001	0.002	0.972
		79	79	84
	LMD		0.330	−0.126
			0.003	0.297
			79	70
	BFT			−0.026
				0.839
				70

BHBA: ketone bodies in blood; LMD: loin muscle depth; BFT: backfat thickness; rs: Spearman correlation for BHBA and the rest of variables; r: Pearson correlation coefficient for variables other than BHBA; *n*: sample size.

**Table 5:** Piglets born alive/litter and % stillborn piglet/litter for gilts and multiparous sows in groups C and T.

Age Group	Variable	Group C		Group T		*p*-Value (Group)
		*n*	Mean ± SD; LS ± SE	*n*	Mean ± SD; LS ± SE	
Gilts						
	Piglets born alive/litter	6	17.83 ± 1.941	6	16.33 ± 4.274	0.452
	% Stillborn piglets/litter	6	8.28 ± 7.964	6	9.99 ± 6.529	0.693
Multiparous sows						
	Piglets born alive/litter	43	16.09 ± 0.543	41	17.39 ± 0.607	0.892
	% Stillborn piglets/litter	43	9.24 ± 0.962	41	4.54 ± 1.076	0.039

Mean ± SD: mean and standard deviation were estimated for gilts. LS ± SE: least squares mean and standard error already corrected by cycle were determined for multiparous sows.

## Data Availability

The original contributions presented in this study are included in the article. Further inquiries can be directed to the corresponding author.
